# Sex and Gender in Ageing and Longevity: Highlights From an International Course

**DOI:** 10.37825/2239-9747.1049

**Published:** 2024-02-27

**Authors:** Giuseppina Candore, Giulia Accardi, Anna Aiello, Giovannella Baggio, Tiziana Bellini, Vittorio Calabrese, Anna P. Carreca, Ignazio Carreca, Anna Masucci, Monica Cattaneo, Serena Dato, Danilo Di Bona, Luca Fabris, Caterina Gambino, Gabriele Di Lorenzo, Claudio Franceschi, Mattia E. Ligotti, Maria C. Manfrinato, Annibale A. Puca, Martina Tamburello, Roberta Vassallo, Calogero Caruso

**Affiliations:** a Laboratory of Immunopathology and Immunosenescence, Department of Biomedicine, Neurosciences and Advanced Diagnostics, University of Palermo, Palermo, Italy; b Department of Laboratory Medicine, University Hospital “P.Giaccone”, Palermo, Italy; c Italian Center for Studies on Gender Health and Medicine, Padua University-Hospital, Padua, Italy; d University Center for Studies on Gender Medicine, University of Ferrara, Ferrara, Italy; e Department of Neuroscience and Rehabilitation, University of Ferrara, Ferrara, Italy; f Department of Biomedical and Biotechnological Sciences, University of Catania, Italy; g Ri.MED Foundation, Palermo, Italy; h Section of Medical Oncology, Department of Surgical, Oncological and Oral Sciences, University of Palermo, Palermo, Italy; i Department of Biomedicine, Neurosciences and Advanced Diagnostics, University of Palermo, Palermo, Italy; j Cardiovascular Department, IRCCS MultiMedica, Milan, Italy; k Department of Biology, Ecology and Earth Sciences, University of Calabria, Rende, Italy; l Department of Medical and Surgical Sciences, University of Foggia, Foggia, Italy; m Department of Medicine, University of Padua, Padua, Italy; n Department of Internal Medicine, Digestive Disease Section, Yale University, New Haven, CT, USA; o Department of Health Promotion Sciences, Maternal and Infant Care, Internal Medicine and Medical Specialties, University of Palermo, Palermo, Italy; p Institute of Information Technologies, Mathematics and Mechanics, Lobachevsky State University, Nizhny Novgorod, Russia; q Institute of Biogerontology, Lobachevsky State University, Nizhny Novgorod, Russia; r Department of Research, ISMETT-IRCCS Mediterranean Institute for Transplants and Highly Specialized Therapies, Palermo, Italy; s Department of Medicine, Surgery and Dentistry, University of Salerno, Salerno, Italy

**Keywords:** Ageing, Age-related diseases, Epigenetics, Gender, Genetics, Immune responses longevity, Sex

## Abstract

Gender medicine is a multidisciplinary science and represents an important perspective for pathophysiological and clinical studies in the third millennium. Here, it is provided an overview of the topics discussed in a recent course on the Role of Sex and Gender in Ageing and Longevity. The paper highlights three themes discussed in the course, i.e., the interaction of gender/sex with, i) the pathophysiology of age-related diseases; ii), the role of genetics and epigenetics in ageing and longevity and, iii) the immune responses of older people to pathogens, vaccines, autoantigens, and allergens. Although largely unexplored, it is clear that sex and gender are modulators of disease biology and treatment outcomes. It is becoming evident that men and women should no longer be considered as subgroups, but as biologically distinct groups of patients deserving consideration for specific therapeutic approaches.

## 1. Gender medicine, a new paradigm

Gender medicine represents an important perspective for pathophysiological studies and clinical practice in the third millennium. Gender medicine considers sexual dimorphism and, therefore, investigates the influence of sex in the biological sense (related to hormones and sex chromosomes) and gender in the social sense (linked to education and social conventions) on the existing differences between men and women in terms of the etiopathogenesis and pathophysiology of diseases and their prevention, clinical phenotype, therapeutic approach, prognosis, psychological and social impact [1].

In fact, sex and gender are not synonymous. Sex refers to the biological and physical characteristics that distinguish individuals as male or female. These characteristics include reproductive organs, sex chromosomes, and secondary sexual features. Gender is a social and cultural construct that refers to the roles, behaviours, activities, and expectations associated with being male or female in a given society in a specified historical period. It is influenced by culture, social norms, and expectations [1–3]. It is important to note, however, that in common language, the term “gender” is often used more broadly to address the differences between male and female, encompassing either biological or social and cultural aspects.

There is a complex interaction between biological factors (sex differences) and socially constructed factors (gender differences) in ageing and longevity. This latter aspect was the focus of the hybrid course (in-person and digital) “Role of Sex and Gender in Ageing and Longevity” led by Professors Calogero Caruso and Giuseppina Candore, organized within the International School of Medical Sciences directed by Professors Ignazio Carreca and Antonino Zichichi of the Ettore Majorana Foundation and Centre for Scientific Culture in Erice, Sicily, Italy, on November 7 and 8, 2023. Academics and scholars from various backgrounds (biochemists, biologists, geneticists, immunologists, geriatricians, physicians) presented data and discussed ideas on the associations between these processes and their implications for human health and longevity. Some of the crucial points that emerged from the scientific sessions and ensuing discussions are examined below.

The significance of gender medicine stems from the seminal observation that, in the last 50 years, many studies have primarily or sometimes exclusively focused on patients of a single sex. This condition of failure of overlooking female specificities (not only in medicine) has been referred to as the Yentl Syndrome, named after the heroine of Singer’s novel who was forced to disguise herself as a man to attend a rabbinical school [4].

Gender Medicine, however, is not confined to women’s health or men’s health, nor does it solely deal with reproductive functions. Rather, Gender Medicine aims at investigating the distinct features pertinent to epidemiology, pathogenesis, diagnosis, therapy and prevention, in the diseases that variably affect both men and women [5].

Striking examples, derived from common and epidemiologically relevant diseases, as well as treatment strategies, include [6,7]:

- Cardiovascular diseases, primarily studied in men, yet myocardial infarction is the leading cause of death in women, exhibiting distinct symptoms and pathophysiological characteristics.
- Cancer presents different symptoms, pathophysiology, and responses to therapy. For instance, solid and haematological neoplasms result in higher mortality in men and display significant gender differences.
- Dementias disproportionately affect women and entail different biological risk factors.
- Osteoarthritis, causing a high degree of motor disability, is prevalent in women.
- Osteoporosis, traditionally studied mainly in women, affects also men but arising about 10 years later, with significant complications and higher mortality following fractures.
- Organ transplants are greatly influenced by sex matching.
- Depression is more frequent in females but often underdiagnosed in men, who have a notably high suicide rate.

Many other examples are worth of mention, as data have been generated in all the fields of medicine. Longevity provides one of the most glaring models to investigate the gender effects.

In 2022, life expectancy at birth in Italy was 80.48 years for men and 84.78 years for women [8]. However, while women tend to live longer, in the last five years of life, they often experience diseases and disability (see paragraph on age-related diseases). In contrast, men facing health problems are more likely to succumb to those problems compared to women with similar health issues [9].

It is, therefore, imperative the transition from the concept of Gender Medicine to that of Gender-specific Medicine, as all medical specialties should be tailored to gender differences. In the era of knowledge of the human genome, Artificial Intelligence, and Precision Medicine, missing Gender-specific Medicine represents a significant gap in global scientific medical awareness. Gender-specific medicine emerges as a paradigm shift at the beginning of the third millennium [7].

Having two X chromosomes instead of an X and Y, entails significant consequences indeed. As early as 2011, the NIH recommended to the scientific community to capture differences and underline similarities in all human diseases affecting both sexes, by inciting studies on gender/sex differences also at the cellular level. Additionally, there is an emphasis on studying gender differences starting from birth, and eventually, from conception [10].

In Italy, since January 2018, the first world law on gender medicine has been enacted, mandating changes in training, information, research, and medical practice [11]. Recently, a Training Plan was signed by the Minister of Health and the Minister of University and Research in April 2023, calling in action the responsibilities from universities, political Regions (the hub of the healthcare system in Italy), Scientific Societies, Professional Associations, Health and Care Professions Council, and Scientific Foundations. Conceivably, gender-specific medicine is not simply a scientific duty, but rather an ethical, moral, social, and legal commitment [12].

## 2. Age-related diseases

Sex contributes to significant differences in the incidence and prevalence of various age-related diseases, and sex-specific mortality rates follow different trajectories during ageing, as evidenced by a clear prevalence of women over men, with substantial variations in the ratio between female and male centenarians (the lowest ratio is reported in Sardinia, Nuoro province, where it is 2:1) [13,14].

The interaction of gender/sex with the pathophysiology of frailty and age-related diseases has been a subject of intense debate in the last ten years, with some important nuances still left to be appreciated. Despite their longer life expectancy, women may face a higher risk of physical and cognitive decline, leading to increased dependency and disability compared to men. This paradox highlights the ambiguous relationship between longevity hand quality of life in the two sexes/genders, emphasizing the need for a deeper understanding of the underlying factors contributing to these differences. Sex chromosomes, hormones, chronic inflammation, immunosenescence, are currently addressed as the major determinants of sex difference in ageing trajectories, influencing the different incidence in age-related diseases in men and women. However, a reconsideration of the paradox was recently proposed by many authors, including Gordon and Hubbard [15] and Reid et al. [16], proposing the frailty status as the forefront between healthy and unhealthy ageing, the base to start from to gain a deeper understanding of the factors underpinning the differences in longevity and health span.

From the preliminary studies on frailty, authors reported sex-specific association of frailty index with age and mortality [17], with females accumulating more deficits than men as long as they age; differences were remarkable in particular when passing from the pre-frail to the frail status [18], because in the very beginning women are more resilient than men, showing a higher adaptability to perform under stress thanks to a greater physiological reserve [19].

In the multisystem physiological theory of human frailty proposed by Taylor et al. [20], two individuals of the same chronological age may respond to the same stressor quite differently, with not frail recovering quickly due to a high level of functional ability and resilience with respect to frail individuals, quickly decompensating and never getting back to the former functional baseline level. The ability to identify frailty or predict resilience in a sex-specific way should provide clues about how to optimize health for both. Circulating biomarkers of frailty refer to six different areas (namely, inflammatory, nutritional, endocrine, and immune markers, metabolic, haematological/renal and oxidative markers) [21]. New emerging biomarkers of frailty (for biomarkers of cardiovascular diseases (CVD) and neurological diseases, see below) can be found in miRNAs, which preliminary study in ageing reported that they may influence health status differently in the two genders, likely depending on the cellular and physiological context [22].

Gender differences in frailty cannot be discarded when studying age-related traits, and interventions to slow frailty progression and delay ageing, avoiding undertreatment (*i.e*., physical activity, nutritional regimen, medications, and supplementation) should become sex-specific, targeted to slow frailty progression and delay ageing, avoiding under treatment. This challenge can be addressed by promoting multidisciplinary team working in geriatric medicine, inciting integrated collaborations between experts in the different fields of biogerontology.

Over the past 20 years, biomarkers related to CVD and neurological diseases, which are currently among the leading causes of death worldwide, have gained increasing importance. The ageing population has pushed the boundaries of research on age-and sex-related biomarkers, facilitating early and differential diagnoses that are useful for personalized patient treatment. There is always an interrelationship between sex and gender in health, disease, and medicine [5]. This holds true in disability in non-communicable diseases, which is approached differently in relation to sex (see above).

In CVD, males experience greater disability than females. However, age is a critical factor for females, as postmenopausal women are less protected [23,24]. Neurological diseases also exhibit significant disparities in disability: females are more affected by Alzheimer’s Disease (AD) and Multiple Sclerosis, while males are more affected by haemorrhagic stroke, epilepsy, and Parkinson’s Disease. Three main biomarkers, Paraoxonase-1 (PON-1) in CVD, Matrix Metalloproteases (MMPs) in both CVD and neurological diseases, and Beta-Secretase-1 (BACE-1) in AD have been proposed as indicator of sex-specific disease phenotypes [25–27].

PON-1 hydrolyses oxidized lipids in LDL, preventing their internalization in macrophages and the formation of foam cells. It plays an antioxidant and anti-inflammatory role in CVD. Females are characterized by higher levels of PON-1, which may partly explain their greater protection from oxidative stress and CVD, particularly during childbearing age [25].

MMPs, responsible for extracellular matrix turnover, can be extensively modulated by cytokines and growth factors produced during inflammatory conditions. As anticipated, inflammation acts by disrupting the delicate balance between the activation and inhibition of MMPs in most CVD. Within the central nervous system (CNS), MMPs play a role in neurogenesis, axonal guidance, and synaptic plasticity, as well as in neuroinflammation, neurodegeneration, and cerebrovascular disorders. There is a clear disparity in MMP levels between males and females in various cardiovascular and neurological disorders [26].

BACE-1 is crucial for generating all monomeric forms of amyloid-β (Aβ), including Aβ42, which aggregates and likely initiates toxicity in AD. Growing evidence suggests that the activity and concentration of BACE-1 in serum seem to reflect those in the brain. Female and male brains follow profoundly different trajectories as they age; female brains undergo age-related changes much earlier than male brains. Early changes in female brains signal the onset of an AD risk phenotype, and women over the age of 70 face a higher risk of elevated sBACE1 activity [27].

In general, a sex and gender approach should always be considered in medicine to have more correct controls and thus deliver more reliable results. Sexual differences, exacerbated further by age progression, induce changes in clinical manifestation, disease progression, and prognosis in both CVD and the CNS. The importance of discovering new sex-specific biomarkers lies in the fact that just because a difference is not obvious, this does not mean it is not significant. Biomarkers, in this context, could offer crucial insights into the mechanisms underlying sex and gender differences in age-related diseases, potentially paving the way for more personalized and effective treatments.

Mitochondria play a crucial role in cellular ageing and lifespan extension, although further studies are needed to understand optimal bioenergetic mechanisms for promoting aerobic energy production and the potential harmful effects of reactive oxygen species (ROS) produced. The generation of ROS is a highly regulated process controlled by a complex network of intracellular signalling pathways. By sensing the intracellular energy and nutritional status, the functional state of mitochondria, and the concentration of ROS produced in mitochondria, the longevity network regulates lifespan across species by coordinating the flow of information along its converging, diverging, and multi-branched signalling pathways. That include vitagenes, which are genes involved in preserving cellular homeostasis during stress conditions [28,29].

Some examples of vitagenes are given below. Heat Shock Proteins (HSPs) are a family of proteins involved in the cellular response to stress, including thermal stress. Hsp32 and Hsp70 are vitagenes belonging to this family. Glutathione is a tripeptide composed of three amino acids, glutamic acid, cysteine, and glycine. It is a potent endogenous antioxidant, which is key in the defence against oxidative stress, contributing to the maintenance of cellular redox balance. Gamma-glutamylcysteine (γ-GC) is the immediate precursor of GSH, and γ-GC ligase is the rate-limiting enzyme in the Meister cycle. The Meister cycle is a biochemical process involved in the biosynthesis of GSH and is therefore central in the regulation of GSH biosynthesis and cellular redox state. Thioredoxin is involved in the regulation of cellular redox status and defence against oxidative stress. The sirtuins are pivotal in the regulation of metabolism and cellular response to stress [30].

NF-E2-related factor 2 (NRF2) plays a key role in maintaining cellular homeostasis by regulating enzymes and proteins involved in sulfur-utilizing redox reactions. Nrf2 contributes to redox homeostasis and functions as an anti-inflammatory agent in various degenerative disorders. So, NF-E2- related factor 2 (NRF2) plays a key role in maintaining cellular homeostasis by regulating enzymes and proteins involved in sulfur-utilizing redox reactions. Nrf2 contributes to redox homeostasis and functions as an anti-inflammatory agent in various degenerative disorders. So, Nrf2-dependent pathways in cellular stress response, along with their target antioxidant vitagenes, are emerging as robust systems capable of preserving redox homeostasis under environmental and metabolic stresses. It is important to note that sexual differences in oxidative stress have been observed in numerous basic and clinical studies, where males exhibit higher oxidative stress compared to females. Sexual differences in oxidative stress persist and worsen with ageing, both in animal and clinical conditions, with males penalised by higher levels of stress compared to females [29,31].

So, special attention is warranted to unveil gender-specific features of neurocognitive deficit associated with ageing and neurodegenerative disorders. Researchers are interested in developing preventive and pharmacological agents to induce optimized stress responses within the hormetic framework. This strategy aims to address chronic degenerative diseases and slow the ageing and age-related neurodegenerative processes on a broader scale. However, dietary polyphenols, acting as hormetins, activate vitagenes, improving intracellular antioxidant defence systems against ROS damage and ameliorating brain health and longevity processes. These effects may also exhibit sex-specific phenotypic patterns [29,32].

In the field of Oncology, apart from cancer peculiar to both sexes, the most recent data show differences in the appearance of oncological diseases common to males or females, in the molecular and immune features, in the response to therapies, as well as in the adverse events associated with them. From this new angle of view, it immediately becomes clear that “gender”, a factor that has so far been little considered in the oncology field, can instead have a strong impact on the prevention, screening, diagnosis, and treatment of these diseases [33]. Lung cancer is paradigmatic in this respect. For years, this disease has been regarded as prerogative of the male sex, especially due to the predominance of tobacco smoking in men. This condition changed significantly during the 1980s when epidemiological data clearly started to show an exponential increase in its incidence in the female population. In those years, indeed, squamous cell carcinoma (SCC) was the most diagnosed type of lung cancer and associated with the male sex. Nowadays, the incidence of adenocarcinoma (AC) has increased significantly, and AC has replaced SCC as the most common type of not SCLC, going from 20% in the 1970s to over 40% in the new millennium [34].

The gap between the two sexes has progressively narrowed mainly due to the alarming increase in smoking habits among women over the last 60 years. Women smokers, even with lower rates of tobacco use, are more likely to develop lung cancer than men. One possible contributing factor to this observation is that female sex hormones such as oestrogens can exacerbate the carcinogenic effects of tobacco. In fact, by inducing the CYP1A1, a phase 1 enzyme expressed by alveolar cells, which metabolizes tobacco-related carcinogens, as polycyclic aromatic hydrocarbons, they lead to an increased generation of highly reactive intermediates, resulting in an increased level of DNA adducts and thereby favouring carcinogenesis. A further mechanism sees CYP1A1 induced by cigarette smoke, capable of metabolizing endogenous oestrogens into potentially carcinogenic forms, such as catechol and quinine [35,36].

Regardless of tobacco smoke, other genetic and environmental factors may contribute to the increased susceptibility of women to get sick of lung cancer. This observation is well exemplified by the lung cancer affecting never or former smokers, which stands as the seventh leading cause of cancer death worldwide. Recent data indicate that about 60% of lung cancer cases occur in former or never smoker patients, which are histologically typified as ACs. Most of these patients are female, with a much higher diagnosis in women than in men (53% of lung cancer in females and only 15% in males) [37]. Expression of oestrogen receptors (ER) by AC cells may partially explain this unique sex association. Moreover, the β isoform of ER is expressed in almost 90% of non-small cell lung cancer (NSCLC) samples, while women with ERβ-negative lung cancer had a small reduction in mortality compared to those with ERβ-positive cancer; in men, however, it is the exact opposite, indicating ERβ as a possible tool for the prognosis of NSCLC in males [38].

The studies summarized in this chapter indicate that there is growing evidence to support the notion that there are significant sex differences in the risk of developing lung cancer, even beyond the steadily increasing smoking habits in women and the enhanced predisposition to the carcinogenic effects of tobacco in women compared to males. That said, the significance of sex and hormonal status as separate contributory factors must be considered in the prognosis and therapeutic management of lung cancer.

These observations provide a solid foundation for launching further efforts dealing with healthcare organization, therapy, biology of cancer, communication, and social interventions, which can be recollected under the vast topic of “Gender Oncology”.

## 3. Epigenetics and genetics

A complex interplay of environmental (*i.e*, epigenetics), historical, and genetic factors, variably interacting in the different parts of the world, appears to play a crucial role in determining the gender-specific probability of achieving longevity. However, on average, male centenarians are healthier than female centenarians, indicating the so-called health-survival paradox: women live longer but are frailer (see previous chapter on age-related diseases). Nevertheless, there is also a different effect of epigenetic and genetic factors on the probability of reaching the extreme limits of human lifespan of males and females although overlooked [13,14,39–41].

Term epigenetics encompasses heritable modifications not stemming from changes in DNA sequence. These modifications influence the individual phenotype by regulating gene expression and activity [42]. Specifically, DNA methylation and histone modification, involving covalent and non-covalent changes to DNA and histone proteins, respectively, modify DNA accessibility and the whole chromatin structure, thereby governing gene expression patterns [43].

More recently, the field of epigenetics has expanded to include the role of small non-coding RNAs in shaping gene expression levels. Since these processes are influenced by environmental factors, epigenetics is often viewed as a link between the genome and the environment in defining ageing and longevity phenotypes [43].

Males and females exhibit sex-specific expression in a wide range of genes, including metabolic enzymes, which affect both basic physiology and the response to environmental exposures. Sexually dimorphic gene expression is largely a function of endocrine differences between males and females [40].

The significance of epigenetic alterations for healthy ageing and longevity was suggested several years ago by studies on twins, revealing that only 25% of their longevity was linked to DNA sequence. The remaining 75%, unexplained by genetics, was attributed to the influence of non-heritable environmental factors on age-related genetic factors (*i.e*., epigenetics) [44,45]. Ageing process is associated with altered epigenetic mechanisms of gene regulation and the manipulation of these mechanisms is central to the effectiveness of age-delaying interventions [46].

A list of the modifications that occurs during ageing together with the proteins involved in such modifications can help in the identification of therapeutic approaches aimed to restore youth. Information Theory of Ageing explains ageing as caused by loss of epigenetic information due to a faithful DNA repair, where only the disruption on the epigenome counts [47].

Studies performed on centenarians of Cilento are an example of the role of epigenetics. They were recruited and analysed to identify genetic variants able to impact on ageing and age-related diseases. The initial Genome Wide Association Study and further replication attempts in independent populations from USA and Germany identified a 4 polymorphisms haplotype homozygous genotype in bactericidal/permeability-increasing fold-containing-family-B-member-4 BPIFB4 enriched in centenarians [48,49]. Further analysis revealed that the BPIFB4 levels were also increased in serum of centenarians and that the Longevity Associated Variant (LAV) induced BPIFB4 increase in plasma and hypomethylation of BPIFB4 itself [50]. *In vitro* and *in vivo* experiments identified protective effects of LAV-BPIFB4 exposure using gene- and protein-based therapies, pointing to LAV-BPIFB4 as a possible therapeutic tool to prevent and even treat ageing and diseases associated with ageing. LAV-BPIFB4 rejuvenating effects were associated with epigenetic changes that can explain its effects [51]. This study did not look for epigenetic differences between males and females, unlike the two studies reported below.

Consistent difference regarding age-associated DNA methylation changes emerged in these two studies. The first is a meta-analysis of 4 large whole blood datasets where 4 aspects of epigenetic age-dependent remodelling between the two sexes, i.e. differential methylation, variability, epimutations and entropy, were compared. A large fraction (43%) of sex-associated probes undergoes age-associated DNA methylation changes, and a limited number of probes showed age-by-sex interaction. It were experimentally validated 2 regions mapping in FIGN and PRR4 genes and showed sex-specific deviations of their methylation patterns in models of decelerated (centenarians) and accelerated (Down syndrome) ageing. While it did not find sex differences in the age-associated increase in epimutations and entropy, the number of probes having an age-related increase in methylation variability is 15 times higher in males compared to females [52].

More recently, the Study of blood DNA from 729 individuals aged 14 to 94 using over 450,000 methylation sites per sample at single-nucleotide resolution (Illumina Infinium Human-Methylation450K BeadChip) showed that most of the CpG sites, for which methylation changes with age were revealed in both sexes, were associated with the genes responsible for the development and functioning of the nervous system. Moreover, in males, unique age-related methylation changes affected CpG sites associated with changes in the immune system and lipid metabolism, as well as the biological functions (KEGG metabolic pathway analysis) of the glutamatergic system, while in females changes involved in gene transcription and translation, and in biological processes involving genes responsible for the development of diabetes or associated with cAMP signalling cascades emerged [53].

Sexual dimorphism influences molecular pathways and networks leading to the establishment of lifetime differences between males and females, and sex-specific epigenetic modifications contribute to these differences. These differences between males and females also extend to the onset and progression of various age-related diseases such as cardiovascular diseases, neurodegenerative disorders, and various types of cancer. Therefore, besides genetics, epigenetics plays a role in establishing gender-specific differences in the susceptibility and severity of age-related diseases [41], previously discussed.

Regarding genetics, in a recent case-control study conducted on subjects of Han Chinese ethnicity, including 564 males and 1614 females aged 100 years or older, and a control group of 773 males and 1526 females aged 40–64 years, sex-specific genome-wide association analyses and sex-specific polygenic risk score analyses on longevity revealed significant and substantial differences in genetic associations with longevity between men and women. The results emphasize how previous genome-wide association studies on longevity, while identifying some sex-independent genetic variants, overlooked sex-specific longevity loci and related pathways. These findings provide a strong contribution to bridge gaps in knowledge, but further studies are sorely needed. The outcomes of such studies could substantially contribute to personalized healthcare and lay the basis for more effective and targeted health interventions for older individuals of both sexes. For instance, the sex-specific loci and pathways found to have significant associations with longevity in this study could be considered as potential contenders for sex-specific genomic biomarkers. These markers could be further explored in comprehensive approach aimed at enhancing personalized health promotions and interventions [54].

It is important to emphasize, however, that many strategies can be employed to achieve longevity, likely because of combinatorial interactions between different factors of the genome and the environment. The complexity of genome-environment interactions must be considered from an evolutionary and ecological perspective. Hence, the concept of “risk allele” is highly context-dependent, changing not only with gender but also with time and geography. Therefore, genetic determinants of longevity are dynamic and depend on the environmental history of a given population. Indeed, it is believed that population-specific genes play a more significant role in attaining longevity compared to those shared among different populations [55,56].

A paradigmatic example is that of interleukin (IL)- 6. Indeed, it had been demonstrated that genetic predisposition to produce high levels of IL-6 is detrimental for longevity in males [57]. However, a meta-analysis [58] conducted on case-control studies did not support a predominant role for the IL-6 −174 polymorphism in achieving longevity across European populations. In fact, there is an indication that male carriers of the GG polymorphism in Italy have a two-fold reduced chance of reaching the centestatus, although this was not observed in other European groups, suggesting a possible interaction between genetics, sex, and environment in achieving longevity.

## 4. Immune responses

In this chapter, we discuss the interaction of gender/sex with the immune responses of older people to pathogens, vaccines, autoantigens, and allergens. In fact, an intricate web of interactions involving biological factors, sex disparities, and socially constructed factors, gender variances, have been emerging in the context of immune ageing [1,59].

In older people, numerous alterations in both innate and acquired immunity have been delineated and frequently considered detrimental, leading to the term immunosenescence. Immunosenescence is an intricate process involving multiple reorganizational and developmentally regulated changes, rather than a simple unidirectional decline in overall function. However, certain immunological parameters are commonly markedly reduced in older people, while robust function tightly correlates with health status. Immune ageing is profoundly influenced by individual immunological history. Indeed, comprehensive studies have allowed the delineation of the determinants of immune system variance, encompassing genetic and environmental factors, sex, smoking, and cohabitation. Age is just one aspect of the immunosenescence challenge. Everyone undergoes a unique ageing process due to individual genetics and life experiences, a concept that applies even more to the immune system, *i.e*., immunobiography [59].

While the impact of biological distinctions between men and women on various aspects of immune responses has long been acknowledged (refer to the details below), it is crucial to recognize that gender, encompassing the social and cultural roles and expectations associated with being male or female, also significantly affects these processes. Gender has the potential to either accelerate immune ageing or promote longevity. By acknowledging the influence of both biological and social factors, it is possible to attain a comprehensive understanding of why men and women undergo divergent trajectories in immune ageing and experience varied outcomes in terms of longevity. Discrepancies in perceived roles of the sexes, both within families and workplaces, contribute to distinct patterns of antigen exposure. Furthermore, variations in micronutrient intake and access to preventive healthcare facilities may exist. Health promotion knowledge frequently correlates with educational attainment, which is unevenly represented between males and females in many cultures and across generations in the Western world. In countries without a universal healthcare system, healthcare access relies on family prioritization strategies to cope with economic constraints, potentially restricting access to specific treatments and adversely affecting immune responses. Consequently, both biological factors and social and behavioural factors associated with gender contribute to disparities in immune responses, susceptibility to infections, autoimmune diseases, and vaccine responses among older individuals [1–3].

It is widely acknowledged that the strength and nature of immune responses varies between males and females. Oestrogens exert immunoenhancing and anti-inflammatory effects, while androgens and progesterone have immunosuppressive effects. On the other hand, immune response is also regulated by genetics. Specifically the X chromosome encodes about 1100 genes, most of which are distinct from the fewer than 100 genes expressed on the Y chromosome that encodes a set of inflammatory pathway genes exclusively expressed in men. Additionally, a high concentration of immune-related genes is located on the X chromosome, specifically those associated with Toll-like receptors (TLR), cytokines, and the activity of T and B cells. To compensate for differences in gene copies, female cells undergo X chromosome inactivation, silencing one copy of the X chromosome permanently. In some individuals, this process may be incomplete, leading to an overexpression of genes on the X chromosome. In the context of incomplete X chromosome inactivation, females may experience alterations in the expression of X-linked genes that promote inflammation and subsequent autoimmunity, such as TLR7/8 [1,2,60].

However, age-related increase in NK cells and CD8^+^ memory T cells, as well as the loss of naive T cells, are similar between men and women. The hallmarks of immunosenescence, including the decline of naive cells, the increase of memory cells, and inflamm-ageing, do not differ basically between older men and women. Nevertheless, there is observed a 15-fold increase in the activation of monocyte-specific loci in men compared to women. With age, specific B cell loci/genes are moderately activated in females but significantly inactivated in males. Consequently, older men exhibit higher genomic activity for monocytes and inflammation, while older females display higher genomic activity for cells of the acquired arm of the immune response. Based on these findings, men are more susceptible to many infections, as evident in the COVID-19 pandemic, while older females demonstrate greater resilience to infections. However, women are more prone to diseases with enhanced immunopathological impact, whether infectious or autoimmune (see below). Therefore, it is not surprising that women tend to live longer than men, and the number of female centenarians exceeds that of male centenarians [3,59,61].

**Fig. 1:**
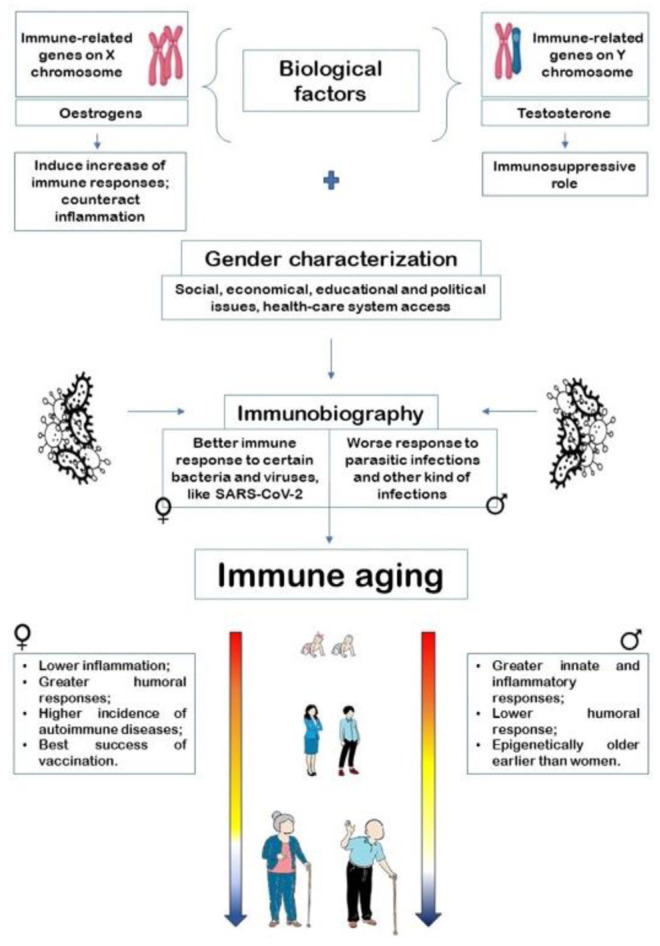
Interaction of sex and gender in defining immune ageing through immunobiography assessment (published under a Creative Commons CC-BY license from Ref. [1]).

Fig. 1 shows the interaction of sex and gender with immune responses in defining immune ageing.

To gain insights into the role of the immune system in extreme longevity, Tαβ, Tγδ and NK immunophenotype of eight semi-centenarians (105+ years) and supercentenarians (110+ years) have been analysed, by flow cytometry, in a Sicilian cohort of 28 women and 26 men. This is because oldest centenarian immune systems is believed to possess unique characteristics that enable them to achieve extreme longevity in a relatively healthy state [62–64].

Concerning Tαβ, the eight semi- and supercentenarians exhibited the lowest percentages of naïve T cells due to their age and the highest percentages of terminally differentiated T effector memory cells re-expressing CD45RA (T_EMRA_), based on their Cytomegalovirus (CMV) status. They also displayed elevated levels of serum pro-inflammatory parameters, although their means were lower than those of the remaining 90+ donors. Some of them demonstrated CD8 naïve and T_EMRA_ percentages and exhaustion/pro-inflammatory markers comparable to the younger ones [62]. In a further study on Tγδ [63], the most noteworthy data involved T_EMRA_, showing a significant increase in Vδ1 cells. The highest values were observed in the oldest centenarians, although with considerable heterogeneity. Finally, in the same sample, a highly significant age-related increase in CD56+CD16+ NK cells has been demonstrated, with the highest values observed in the oldest centenarians, albeit again with considerable heterogeneity [64].

These studies support the notion that immune ageing, especially in the oldest centenarians, demonstrates considerable variability not attributable to a single factor but rather the result of a combination of several factors. As previously stated, everyone ages differently due to their uniqueness in genetics and life experiences, and this holds particularly true for the immune system, as everyone has had a different immunological history [59].

Furthermore, the findings on inflammatory markers, Tαβ and Tγδ T_EMRA_, CD16+CD56+ NK, and CMV seropositivity in centenarians, when considered in the context of the latest literature, suggest that these changes might not be unfavourable for centenarians, especially the oldest ones. Indeed, CMV is responsible for a substantial subset of Tαβ effector memory virus-specific cells, and many of these are T_EMRA_. These cells are perfectly equipped to control the virus without further T-cell expansion [65]. Moreover, *in vivo* γδ T_EMRA_ expansion has been demonstrated to correlate with a reduced risk of cancer onset or leukaemia recurrence, as well as with the clearance of CMV infection, in allogeneic stem cell recipients and kidney transplant patients, respectively [66]. Finally, it has been demonstrated that the percentage of circulating CD16+CD56+ NK cells is negatively correlated with the occurrence of colorectal cancer and its staging [67]. Therefore, again, these increases should not be considered unfavourable for the oldest centenarians.

Regarding the role of sex, it is important to emphasize that this cohort included only one male semi-supercentenarian. This imbalance is reflective of the fact that women are statistically more likely to achieve exceptional longevity [3,68]. Therefore, some observed differences in the distribution of cellular subsets between males and females have been influenced by the disproportionate representation of the oldest centenarian women.

Autoimmune diseases encompass more than 80 chronic disorders that affect nearly 5% of the population in Western countries. They are characterized by an exaggerated immune response leading to damage and dysfunction in specific or multiple organs and tissues. Autoimmune diseases are typically more prevalent in women than in men and are considered the fourth leading cause of disability for women [69].

The reduction of microbial load during childhood, occurring in the Western world, leads to decreased stimulation of Treg cells, a specialized subset of T lymphocytes capable of suppressing the activation of the immune system. This results in reduced control of both Th1 responses, which are cell-mediated, and Th2 responses, which are antibodymediated, primarily of the IgE type. These are responsible, respectively, for the increased prevalence of autoimmune diseases and allergic conditions in the Western world [70,71].

The development of autoimmunity and the progression to autoimmune diseases result from the interaction over time between genetic, epigenetic, and environmental factors. Genetic factors and epigenetic alterations predispose individuals to the loss of tolerance, subsequent development of autoantibodies, tissue damage, and the onset of autoimmune diseases. Environmental factors are less well-known, but they serve as the “triggers” that initiate and promote disease progression [72,73].

The strong sex bias in susceptibility to autoimmune diseases may stem from the distinct effects of sex-specific hormones in contributing to the disease and their varied roles in relation to reproductive status. Oestrogens, found in elevated levels during pregnancy, influence immunity by modulating lymphocyte development and function, and promoting cytoprotection. These actions of oestrogens could either enhance cell-mediated disease or exacerbate antibody-mediated disease. Progesterone and androgens exhibit anti-inflammatory and immunosuppressive actions, respectively, generally beneficial in autoimmune diseases. Prolactin, elevated during pregnancy, induces pro-inflammatory effects and tends to worsen autoimmune disease [74].

Oestrogens enhance both cell-mediated and humoral responses. Concerning B cells and antibody production, oestrogens promote B maturation, isotypic switching, survival, and increase antibody production. They also affect the Th1/Th2 and Th17/Treg ratio. In contrast, androgens downregulate immune response and antibody production, promote tolerance, and suppress T differentiation. However, the influence of sex hormones is associated with their levels and, consequently, reproductive function. If B cells play a central role through antigen presentation, autoantibody production, and/or cytokine secretion, 17-beta oestradiol (E2) is likely to accelerate the onset of disease in the early reproductive years. If T cells play an equal or more significant role than B cells, the onset of the disease in women may be delayed because E2 inhibits T cell autoimmunity but stimulates B cell autoimmunity. In this situation, the onset of the disease could shift to the late reproductive phase or postmenopause [1,2].

Closely related to pregnancy is microchimerism, which refers to the trafficking of cells from the foetus to the mother and vice versa. It has been proposed that foetal microchimerism plays a beneficial role in aiding a woman’s recovery from an injury by providing additional multipotential stem cells that could be utilized to repair damage. In contrast, maternal microchimerism can be harmful since maternal cells are a potential source of graftversus-host response, as observed in systemic sclerosis. Given that microchimerism occurs during pregnancy, it may contribute to sex differences in autoimmune diseases [74].

Concerning menopause and ageing, it has been observed that there is a progression of disease in women with rheumatoid arthritis. However, ageing and menopause lead to a decrease in the progression of Systemic Lupus Erythematosus (SLE) in women. An earlier onset of menopause was correlated with an increased likelihood of developing rheumatoid arthritis and SLE [75].

Regarding the role of the environment in sexual dimorphism in autoimmunity, a substantial body of scientific evidence indicates that genetic variability alone cannot account for the variability in the risk of developing chronic diseases, including most autoimmune diseases. The environment, interacting with the genotype, may influence the prevalence and risk of developing an autoimmune disease or affect the severity of the disease. Men and women are exposed to environmental factors to varying degrees. Additionally, they exhibit different physiological responses to such factors. This differing exposure and response might impact the prevalence and risk of developing an autoimmune disease or the severity of the disease between men and women. Endocrine-disrupting chemicals act through multiple mechanisms, demonstrating both estrogenic and anti-estrogenic properties, reducing androgen production, and influencing epigenetic regulation. This exposure is nearly unavoidable in contemporary societies, as these compounds can be found in drinking water, cosmetic products, paper products, and food and beverage containers. Distinct male-female responses to exposure could contribute to the dysregulation of the immune system to varying degrees, depending on the specific disease [76].

In recent years, several studies have emphasized the role of the microbiome in the pathogenesis of autoimmune diseases, as the loss of immune tolerance can be triggered by changes in microbial composition, known as dysbiosis. Host genetic susceptibility, hormones, and various extrinsic factors, such as specific drug intake, unhealthy diets, inappropriate microbial exposure, childbirth delivery, or breastfeeding, may induce alterations in the composition of the gut microbiota. Reduced richness and disturbances in the taxonomic commensal and metabolite composition have been widely associated with the development of multiple autoimmune inflammatory disorders [77].

In conclusion, in women with a genetic predisposition to autoimmune diseases, external environmental stimuli influence modifying factors as well as endocrine transitions via epigenetic mechanisms. Additionally, there are interactions between hormones, on one hand, and the interplay between Th1 and Th2 immune responses on the other. Both of these phenomena, endocrine and immune response, are influenced in various ways during the female transition states, depending on the circulating concentrations of different hormones and cytokines, which may be regulated by epigenetics. Thus, hormonal fluctuations, immune polarization, and transition states collectively propel susceptible women over the autoimmune tipping point, leading to the manifestation of overt clinical disease [78].

In the past, allergy was considered a minor issue in older people. However, recent series of epidemiological studies indicates that allergic diseases are more prevalent than expected among older adults. Additionally, they have a significant impact on quality of life and socioeconomic costs. Moreover, there is evidence of the importance of sex/gender differences in allergic diseases and their treatment.

Sex and age play a role in determining the presence or severity of allergy. Allergic diseases are more frequent in women from 18 to 65 years, while under age 18 or over 65 it is more prevalent in males. This might be due to sexual hormone effect. Accordingly, fluctuations of hormones during puberty, menstruation, pregnancy, and menopause, alter asthma symptoms and severity. Asthma attacks are, indeed, more frequent during the perimenstrual days. Although no age-related increase in IgE has been demonstrated, both total and specific IgE tend to decrease after menopause and increase in women using hormone replacement therapy. Collectively, these data suggest a significant impact of sexual hormone on immunity resulting in different atopic responses. Experimental evidence confirms these findings. Transgenic male mice expressing aromatase have low testosterone levels and high oestradiol levels, and this correlates with IgE levels, greater in aromatase mice compared to their wild type counterparts. Conversely, female mice knock-out for aromatase have low oestradiol levels [79–82].

Immediate hypersensitivity (Type I) is the most prevalent immunological disorder, affecting approximately 25% of the population in industrialized countries. Type I reactions manifest with symptoms ranging from a diminished quality of life to severe life-threatening conditions, including eczema, conjunctivitis, rhinitis, asthma, and anaphylaxis. The escalating prevalence of allergies can be attributed to factors such as climate changes, pollution, a Westernized diet, and alterations in the microbiota. The microbiota plays a pivotal role in initiating and sustaining immunoregulatory circuits and tolerance. Modifications to the microbiota can result in immune dysregulation, leading to a low-grade chronic inflammatory state [83].

Most studies investigating allergic diseases and their underlying mechanisms have primarily focused on children or adolescents rather than adults over the age of 65, who are expected to constitute approximately 25% of the population in industrialized countries in the coming years, owing to global demographic changes and increased life expectancy. Given the constant rise in the older population, there is a growing need for diagnostic and therapeutic programs specifically tailored to older patients. Consequently, the increasing prevalence of allergic diseases in older individuals suggests that these conditions will become a significant public health burden soon [83,84].

Allergic diseases in older individuals are influenced by the general ageing of cells, immunosenescence, and typical changes in tissue structure associated with advanced age. Additional contributing factors to the increased prevalence of allergic diseases in older individuals include various comorbidities that may interfere with the development and nature of allergic reactions. Moreover, there is a shift in older individuals from Th1 responses to Th2 responses, thereby favouring allergic reactions. A deeper understanding of the mechanisms underlying immunosenescence and its impact on allergic inflammation is expected to lead to improved therapies. Optimal treatment for older patients necessitates collaboration between the patient, geriatrician, and allergist [83,84].

Age also plays a role in sensitization regardless of gender. Sensitization intensity for common inhalant or food allergens tends to decrease with age, with significant difference among the allergens. Generally, allergy to house dust mites and fish tend to maintain a greater sensitization compared to other allergens [85].

There is an interaction between sex and genes involved in immune response in asthma. Polymorphisms in the Thymic Stromal Lymphopoietin, a cytokine released by respiratory airway epithelial cells upon exposure to allergens, pollutants, viral, fungal, and bacterial components resulting in Th2 cell polarization, have been described. The T allele of rs1837253 was significantly associated with a reduced risk of asthma in males only, whereas the T allele of rs2289276 was significantly associated with a reduced risk of asthma in females only [86–88].

In atopy, metabolic abnormalities in the leukotrienes (LTs) pathway are known to play a crucial role, and sex has been identified as a key variable in LT biosynthesis. Notably, interesting variability in LT production is often associated with single nucleotide polymorphisms (SNPs) in the arachidonate 5-lipoxygenase (ALOX5) gene, which encodes the leukotriene-synthesizing enzyme machinery, 5-lip-oxygenase (5-LO). A recent study established a link between variations in ALOX5 gene SNPs and sex-related differences in leukotriene production within a gender-balanced cohort of atopic subjects. This led to a decrease in serum levels of 5-LO and LTB4 in men and an increase in women levels [89,90].

## 5. Concluding remarks

Gender-specific medicine is an innovative concept in medicine. Its inception in the 1990s quickly underwent significant evolution by adopting an approach aimed at highlighting both gender and sex, as well as the factors that define them, such as biological, environmental, cultural, and socioeconomic factors, as potentially relevant determinants of physiology, pathophysiology, and clinical characteristics of diseases.

In many fields of medicine, sex and gender as modulators of disease biology and treatment outcomes are still largely unexplored. Considering the growing evidence of gender differences in the pathophysiology of various diseases and their treatment, men and women should no longer be regarded as subgroups, but as biologically distinct groups of patients deserving specific therapeutic approaches.

To support further this need, is the observation that the world population is progressively ageing, with dizzying and unpredictable increases especially in the older age groups, and an equally dramatic rise in people aged 100+. Unfortunately, the ageing of the population is not accompanied by an acceptable quality of life, thus creating increasing financial and health problems such as the saturation of hospital facilities by patients with multiple chronic disabling pathologies to the detriment of acute diseases to which the hospital should instead be traditionally dedicated [91].

Many individuals, especially women, indeed age in conditions of physical and mental health deterioration that impinge upon self-sufficiency. Therefore, the continuation of research and education in the field of the role of sex and gender in ageing is of utmost interest, so much so that a second course on the Role of Sex and Gender in Ageing and Longevity has been scheduled for 2024 at the Ettore Majorana Center and Foundation in Erice. Furthermore, given the importance of this topic, most authors of this review, along with other distinguished colleagues, are called to provide a contribution to a book with the same title, edited by Professor Caruso, which will be published by Elsevier at the beginning of 2025.
